# Insights into O-GlcNAcylation and programmed cell death in cancer

**DOI:** 10.3389/fcell.2025.1560491

**Published:** 2025-09-22

**Authors:** Xiaohan Yan, Wenhao Ren, Zhuang Zhu, Shaoming Li, Rui Shi, Kai Sun, Keqian Zhi, Ling Gao, Jingjing Zheng

**Affiliations:** ^1^ Department of Oral and Maxillofacial Reconstruction, The Affiliated Hospital of Qingdao University, Qingdao, China; ^2^ School of Stomatology, Qingdao University, Qingdao, China; ^3^ Department of Oral and Maxillofacial Surgery, The Affiliated Hospital of Qingdao University, Qingdao, China; ^4^ Key Lab of Oral Clinical Medicine, The Affiliated Hospital of Qingdao University, Qingdao, China; ^5^ Department of Endodontics, The Affiliated Hospital of Qingdao University, Qingdao, China

**Keywords:** O-GlcNAcylation, cancer, programmed cell death, apoptosis, autophagy, pyroptosis, ferroptosis, necroptosis

## Abstract

O-GlcNAcylation is an essential post-translational modification that adds O-linked β-N-acetylglucosamine (O-GlcNAc) to numerous proteins’ serine or threonine residues. Several studies have indicated O-GlcNAcylation regulates various processes related to cancer, including signal transduction, transcription, cell division, metabolism, and cytoskeletal regulation. Programmed cell death (PCD) is a regulated and organized form of cell death controlled by genes, including apoptosis, autophagy, pyroptosis, necroptosis, and ferroptosis. As research on PCD has become increasingly in-depth, a potential link between O-GlcNAcylation and PCD has emerged. This review will focus on the complex relationships between O-GlcNAcylation and different PCD pathways, which are closely tied to the onset, progression, and resistance of cancer. By clarifying the relationship between O-GlcNAcylation and PCD, we aim to create a theoretical basis for improving anti-cancer treatments, with promising potential for clinical application.

## Graphical Abstract

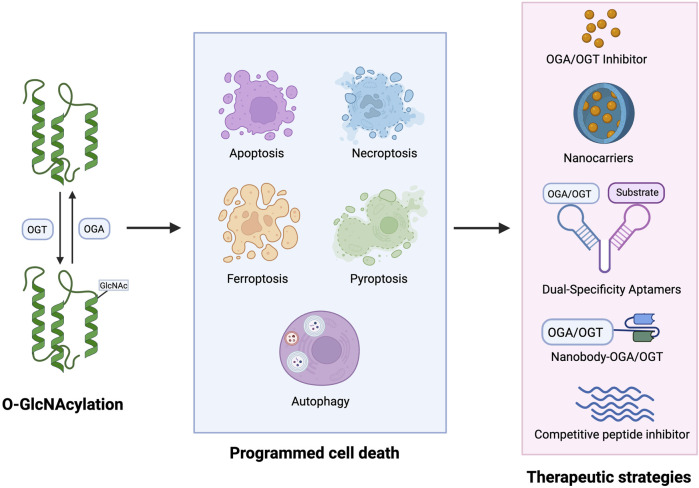

## Introduction

Programmed cell death (PCD) is a crucial terminal pathway for cells in multicellular organisms. It plays a significant role in various biological events, including morphogenesis, tissue homeostasis, and eliminating harmful cells (Sun and Peng, 2009). PCD dysregulation contributes to the pathogenesis of many diseases including cancer (Pilátová et al., 2023). Certain forms of PCD, such as apoptosis, autophagy-dependent cell death, pyroptosis, ferroptosis, and necroptosis, are closely linked to cancer and various other diseases (Wang et al., 2023). Therefore, understanding the mechanisms that regulate PCD is crucial for developing strategies to manipulate it in the management of cancer.

Protein post-translational modifications (PTMs) refer to the covalent alterations made to proteins through the addition of small functional groups or complex biomolecules to specific amino acid residues (Wa et al., 2005). In recent decades, there has been a growing recognition of the critical roles that PTMs, such as phosphorylation, glycosylation, acetylation, and ubiquitylation, play in regulating various cellular processes. As a result, these modifications have attracted considerable interest in the field of molecular biology (Yang and Qian, 2017). O-GlcNAc protein modification, also known as O-GlcNAcylation, is commonly found in the cytoplasm, nucleus and mitochondria. It was first identified on the surface of mouse lymphocytes by Carmen-Rosa Torres and Gerald Hart in 1983 (Torres and Hart, 1984; Hanover et al., 2010). This modification is a type of post-translational modification that involves glycosylation, where a single GlcNAc molecule is attached to the serine or threonine residues on proteins via an O-linked β-glycosidic bond (Holt et al., 1987). Unlike conventional protein glycosylation, O-GlcNAcylation is a dynamic and reversible process. O-GlcNAcylation is highly responsive to a wide range of extrinsic stimuli, including osmotic, oxidative, hyperthermic, and genotoxic stresses (Yang and Qian, 2017; Chatham et al., 2021). These mechanisms for sensing cellular stress are closely related to PCD (Zuppini et al., 2007; Li et al., 2024). However, the role of O-GlcNAcylation in PCD remains underexplored. This review aims to systematically organize O-GlcNAcylation-regulated PCD mechanism and to propose novel strategies for tumor therapy.

## An overview of O-GlcNAcylation

Glycosylation is one of the most common and variable forms of PTMs (Xu et al., 2024). The two primary types of glycosylation are N-glycosylation and O-glycosylation. In N-glycosylation, the core of N-linked glycans is always attached to asparagine residues in the protein backbone. These glycans typically have a common pentasaccharide core consisting of two N-acetylglucosamine (GlcNAc) residues and three mannose residues (Higel et al., 2016). In contrast, O-glycosylation begins with the addition of a single sugar molecule to serine or threonine residues (Calvete et al., 2008). There are several types of protein O-glycosylation. Mucin-type O-glycosylation starts with N-acetylgalactosamine (GalNAc) and forms a diverse glycan chain, which is typically found in mucins and other secreted proteins. O-GlcNAcylation occurs in the cytoplasm and nucleus and plays a role in regulating transcription, metabolism, and the stress response. O-linked fucose (O-fucose) and O-linked glucose (O-Glc) function as parts of the Notch receptor and are involved in regulating the Notch signaling pathway. O-Xylosylation is catalyzed by xylosyltransferase, leading to the formation of a glycosaminoglycan (GAG) chain (Li et al., 2023; Zhang and Ten Hagen, 2019).

**FIGURE 1:**
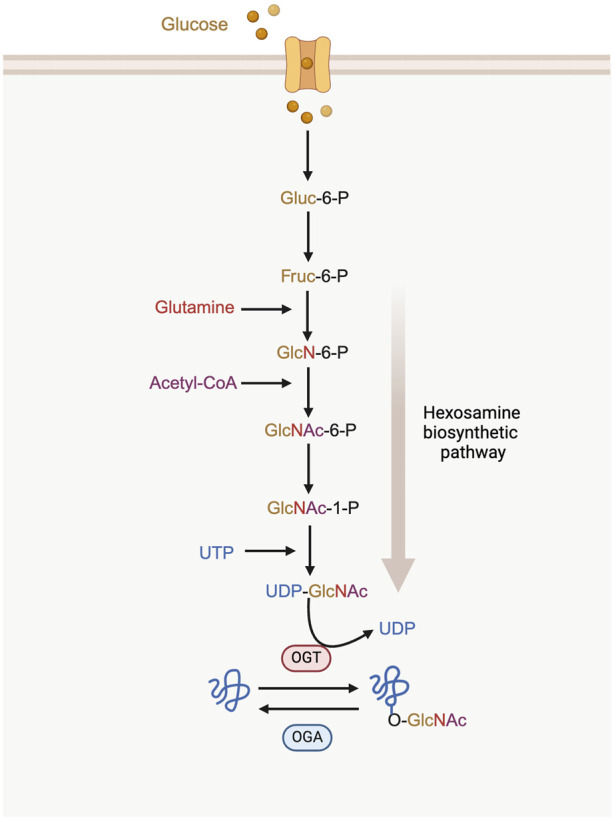
The process of hexosamine biosynthetic pathway (HBP) and protein O-GlcNAcylation. UDP-GlcNAc synthesis requires the incorporation of glucose, glutamine, acetyl-CoA and UTP. O-GlcNAc on proteins is cycled between the addition of OGT and the removal of OGA. Gluc-6-P: glucose-6-phosphatase; Fruc-6-P: fructose-6-phosphate; GlcN-6-P: glucosamine-6-phosphate; GlcNAc-6-P: N-acetylglucosamine-6-phosphate; GlcNAc-1-P: N-acetylglucosamine-1-phosphate.

O-GlcNAcylation acts as a nutrient sensor through the hexosamine biosynthetic pathway (HBP). This pathway is crucial for sensing metabolic status and regulates the production of uridine diphosphate N-acetylglucosamine (UDP-GlcNAc) (Lam et al., 2021). The HBP utilizes metabolites from several key metabolic pathways, including glucose (derived from carbohydrate metabolism), glutamine (from protein and amino acid metabolism), acetyl-CoA (from lipid and fatty acid metabolism), and uridine triphosphate (UTP) (from nucleic acid and nucleotide metabolism) to produce the uridine diphosphate N-acetylglucosamine (UDP-GlcNAc) (Paneque et al., 2023). This nucleotide sugar is the substrate of protein O-GlcNAcylation (Figure 1) (Tran and Wang, 2019).

A single N-acetylglucosamine (GlcNAc) moiety is attached to the hydroxyl oxygen atom of serine or threonine residues on proteins. This modification is catalyzed by O-GlcNAc transferase (OGT), while its removal is carried out by O-GlcNAcase (OGA) (Torres and Hart, 1984).

Synergistic interactions between OGT and OGA are critical for maintaining O-GlcNAcylation levels within an optimum range (Ciraku et al., 2022). Additionally, fluctuations in the availability of UDP-GlcNAc also affect O-GlcNAc levels. When the levels of glucose, glucosamine or free fatty acids increase, or when the key enzymes of the HBP are overexpressed, UDP-GlcNAc levels rise correspondingly. This increase ultimately leads to a higher overall level of O-GlcNAcylation in intracellular proteins (Lazarus et al., 2011; Ong et al., 2018).

Altered O-GlcNAcylation has been observed in cell lines of various cancers (Slawson and Hart, 2011; de Queiroz et al., 2014; Trinca and Hagan, 2018). One potential explanation for this phenomenon is a change in the metabolic state of the cells, shifting from oxidative phosphorylation to aerobic glycolysis, a process known as the Warburg effect (Pavlova and Thompson, 2016). In cancer cells, excess glucose primarily enters glycolysis, which increases the flow toward alternative glucose pathways, such as HBP (Ouyang et al., 2022). In addition, imbalanced enzymatic activity of OGT and OGA due to somatic mutations or altered protein stability (Tang et al., 2023; Peng et al., 2021) is also a contributing factor.

Imbalanced O-GlcNAcylation can crosstalk with other PTMs to promote malignant tumor progression. O-GlcNAcylation interacts extensively with phosphorylation by regulating the phosphorylation of adjacent residues or competing for the same serine or threonine residues (Hart et al., 2011). This interaction has been shown to regulate the activation of AMPK and alter the substrate selectivity of OGT in several cell lines (Bullen et al., 2014), potentially affecting cellular gene expression, cell growth, and apoptotic cell death (Hart et al., 2011). For instance, the O-GlcNAcylation of AMPK reduces levels of phospho-AMPK and its activation, which may subsequently decrease the levels of p21 and p27, both of which are cell cycle inhibitors dependent on AMPK, as well as apoptosis in cervical cancer cells (Kim et al., 2019). In addition to phosphorylation, researchers are exploring the complex interactions between O-GlcNAcylation and other PTMs. OGT-mediated O-GlcNAcylation of YTHDF2 on Ser^263^ enhances its protein stability and oncogenic activity by preventing its ubiquitination (Yang et al., 2023). O-GlcNAcylation of SIRT1 at the Ser^549^ site directly enhances the deacetylase activity of SIRT1, protecting cells from stress-induced apoptosis (Han et al., 2017).

## OGT and OGA

OGT is encoded by a single gene in Xq13 of the human genome. This genome is spliced and translated into three distinct isoforms: nucleocytoplasmic OGT (ncOGT), mitochondrial OGT (mOGT), and short OGT (sOGT) (Nolte and Müller, 2002). The N-terminus of OGT contains a tetratricopeptide repeat (TPR) domain, which is essential for recognizing and binding to protein substrates (Love et al., 2003). In contrast, the C-terminal domain is responsible for glycosyltransferase activity (Zhang et al., 2022). Opposing OGT is OGA, an enzyme that is predominantly localized in the cytosol, with some presence in the nucleus (Wells et al., 2002). OGA is categorized as a member of CAZY glycoside hydrolase (GH) family 84 (GH84), which includes two major splice isoforms known as long (lOGA) and short (sOGA) (Alteen et al., 2021). The catalytic activity of OGA primarily relies on its N-terminal structural domain (Stephen et al., 2021).

Both OGT and OGA are evolutionarily conserved (Love et al., 2010) and expressed throughout mammalian cells. Their structures have been resolved (Roth et al., 2017; Meek et al., 2021). Early research in mammals has shown that the genetic knockout of OGT leads to embryonic lethality, while the knockout of OGA results in perinatal lethality. This suggests that O-GlcNAcylation is crucial for the development of organisms (Shafi et al., 2000; Yang et al., 2012). Furthermore, dysregulation of OGT and OGA is associated with various pathological conditions (Chatham et al., 2021). Aberrant expression of OGT and OGA are often found in many tumors, suggesting a role in tumor promotion (Liu et al., 2024).

In recent years, the O-GlcNAc modification has emerged as a key regulator of various cellular processes (Martinez et al., 2017; Bacigalupa et al., 2018). However, the potential significance of protein O-GlcNAcylation in mediating both pathological and physiological processes in numerous human diseases—such as cancer, diabetes, neurodegenerative disorders, and cardiovascular diseases—has only recently been reported and remains largely unexplored (Nie and Yi, 2019).

## O-GlcNAcylation and programmed cell death

PTMs significantly affect almost all cellular biological processes. The diversity and crosstalk have been linked to PCD in cancer such as apoptosis, autophagy and ferroptosis (Di et al., 2024; Ai et al., 2023; Liu et al., 2022). Research on PTMs has become a vital focus in cancer studies, aiming to enhance our understanding of cancer biology and to identify new biomarkers and therapeutic targets (Pan and Chen, 2022).

Glycosylation is a key mode of PTMs in living organisms, playing a crucial role in regulating PCD by influencing protein folding, transport, and localization. For instance, N-glycosylation in the α-I domain of integrin plays a pivotal role in collagen and laminin binding. Abolished N-glycosylation results in downregulation of focal adhesion signaling and increased cellular apoptosis (Huang et al., 2021). Additionally, inhibiting N-glycosylation of mTRAIL-R leads to increased formation of the death-inducing signaling complex (DISC) and subsequent activation of caspase-8. Blocking the N-glycosylation of 4F2hc could reduce 4F2hc protein stability and sensitize PDAC cells to ferroptosis (Estornes et al., 2018; Ma et al., 2023).

**FIGURE 2:**
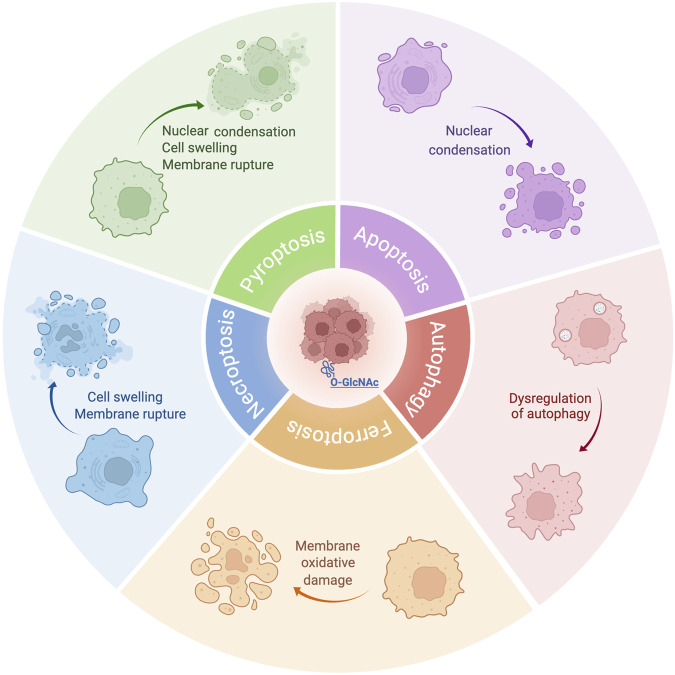
O-GlcNAcylation-mediated five PCDs play a role in cancer. O-GlcNAcylation is an essential post-translational modification that plays a role in cancer by adding O-GlcNAc to numerous proteins’ serine or threonine residues affecting PCD in cancer cells including apoptosis, autophagy, ferroptosis, necroptosis and pyroptosis.

As a dual sensor for nutrient availability and cellular stress, O-GlcNAcylation is highly dynamic (Wells et al., 2002; Vosseller et al., 2001), suggesting that O-GlcNAcylation is closely related to PCD. In this discussion, we will focus on the relationship between five common types of PCD: apoptosis, autophagy, necroptosis, ferroptosis and pyroptosis, and their connection to O-GlcNAcylation (Figure 2). Investigating O-GlcNAcylation may provide new avenues for treating related diseases.

## O-GlcNAcylation and apoptosis

Apoptosis is an ordered and orchestrated cellular process that takes place under both normal physiological and pathological conditions (Wong, 2011). This process is genetically controlled and is primarily categorized into three pathways: endogenous pathways (the mitochondrial pathway), exogenous pathways (the death receptor pathway), and pathways induced by endoplasmic reticulum (ER) stress (Hu et al., 2018). Since increased levels of protein O-GlcNAc were reported to reduce cardiomyocyte apoptosis in 2007 (Champattanachai et al., 2007),a growing number of studies have shown that apoptosis is regulated by O-GlcNAcylation. This modification plays a crucial role in the onset and progression of cancer by either promoting or suppressing apoptosis. In neuroblastoma N2a cells, O-GlcNAcylation inhibits oxidative stress-induced apoptosis by modulating the expression and activity of signal transducer and activator of transcription 3 (STAT3) and forkhead box protein O 1 (FOXO1) (Zhang C. C. et al., 2024). In human lung carcinoma, O-GlcNAcylation of p53/c-Myc regulates cisplatin (CDDP)-induced apoptosis in lung cancer cells. Under conditions of high p53 activation, O-GlcNAcylation of p53 promotes its ubiquitin-mediated proteasomal degradation, leading to an increase in oncogenic and anti-apoptotic functions (Luanpitpong et al., 2017). By contrast, O-GlcNAcylation of c-Myc exerts the opposite effect. Additionally, during liver cancer progression, O-GlcNAcylation of β-catenin increases its expression, stability, and nuclear accumulation, promoting liver cancer cell proliferation while inhibiting apoptosis (Table 1) (Gao et al., 2019).

**TABLE 1:** O-GlcNAcylation-PCD axis in diverse cancers.

Target gene	Type of PCD	Disease models	Biofunction	References
STAT3/FOXO1	apoptosis	Neuroblastoma	O-GlcNAcylation inhibits oxidative stress-induced apoptosis by modulating the expression and activity of STAT3 and FOXO1	Zhang et al. (2024a)
p53/c-Myc	apoptosis	Lung carcinoma	O-GlcNAcylation of p53 leads to an increase in oncogenic and anti-apoptotic functions. O-GlcNAcylation of c-Myc exerts the opposite effect	Luanpitpong et al. (2017)
β-catenin	apoptosis	Liver cancer	O-GlcNAcylation of β-catenin increases its expression, stability, and nuclear accumulation, inhibiting liver cancer cell apoptosis	Gao et al. (2019)
DR4	apoptosis	Gastric cancer	O-GlcNAcylation of DR4 enables both apoptotic and necroptotic tumor cell death	Lee et al. (2019)
SNAP29	autophagy	Glioblastoma, pancreatic ductal adenocarcinomas, etc.	Enhanced the O-GlcNAcylation of SNAP29 leads to the blockage of autophagic flux and cell death	Pellegrini et al. (2023)
ULK1	autophagy	Head and neck squamous cell carcinomas	ULK1 O-GlcNAcylation promotes ULK1 stability and autophagosome-lysosome fusion, which could promote HNSCC survival	Shi et al. (2022)
AMPK	autophagy	Bladder cancer	O-GlcNAcylation of AMPK suppresses the activity of this regulator, thereby inhibiting autophagic flux	Jin et al. (2020)
TFRC	ferroptosis	Hepatocellular carcinoma	O-GlcNAcylation of TFRC at Ser^687^ reduces the TFRC protein level and decreases the resistance of HCC cells to ferroptosis	Zhou et al. (2024b)
TFRC	ferroptosis	Hepatocellular carcinoma	O-GlcNAcylation increases the sensitivity of HCC cells to ferroptosis by enhancing the t the expression of TFRC	Zhu et al. (2021)
ZEB1	ferroptosis	Pancreatic cancers	O-GlcNAc modification of ZEB1 enhances its stabilization and nuclear translocation, thus decreasing ferroptosis	Wang et al. (2022)
p53	pyroptosis	Lung carcinomatous, euroblastoma	De-O-GlcNAcylation of p53 enhances its stability and promotes pyroptosis	Wang et al. (2024)

Interestingly, O-GlcNAcylation is regarded as both an apoptotic inhibitor and an activator, highlighting its contradictory role in regulation. For example, tumor necrosis factor (TNF)-related apoptosis-inducing ligand (TRAIL) is acknowledged for its ability to trigger selective apoptosis in tumor cells (Micheau, 2018). The O-GlcNAcylation of human TRAIL receptor with a death domain, TRAIL-R1 (DR4), enables both apoptotic and necroptotic tumor cell death (Lee et al., 2019). Furthermore, AKT is a well-known key regulator of cell death and survival, and O-GlcNAcylation negatively regulates the activation of AKT signaling, ultimately triggering apoptosis (Table 2) (Shi et al., 2015).

**TABLE 2:** O-GlcNAcylation-PCD axis in no-cancer diseases.

Related gene	Type of PCD	Disease models	Biofunction	References
AKT	apoptosis	Cerebral ischaemia-related diseases	O-GlcNAcylation negatively regulates the activation of AKT signaling, triggering apoptosis	Shi et al. (2015)
ULK1	autophagy	Autophagy-related diseases	ULK1 O-GlcNAcylation leads to the production of PI(3)P, which is necessary for the initiation of autophagy	Pyo et al. (2018)
FTH	ferroptosis	Diseases related to iron overload	De-O-GlcNAcylation of FTH promotes its interaction with NCOA4 and activates ferroptosis	Yu et al. (2022)
RIPK3	necroptosis	Septic inflammation	O-GlcNAcylation of the RIPK3 prevents RIPK3-RIPK3 homo-interaction and inhibited necroptosis signaling	Li et al. (2019)
RIPK3	necroptosis	Alzheimer’s disease	By modifying RIPK3, O-GlcNAcylation suppresses the phosphorylation of RIPK3 and the interaction between RIPK1 and RIPK3	Park et al. (2021)
RIPK1	necroptosis	Erythrocyte necroptosis-related diseases	O-GlcNAcylation of RIPK1 inhibits its phosphorylation at Ser166 and prevents the formation of the RIPK1-RIPK3 complex	Seo et al. (2023)
NEK7	pyroptosis	Osteoarthritis	O-GlcNAcylation of NEK7 induced by OGT enhances chondrocyte pyroptosis through the suppressive interaction between NEK7 and NLRP3	He et al. (2024)
NLRP3	pyroptosis	Periodontitis	LPS induces pyroptosis in HGFs by increasing OGT expression and promoting the O-GlcNAcylation of NLRP3	Zhou et al. (2024b), Yang et al. (2024)
NLRP3	pyroptosis	Non-alcoholic fatty liver disease	BPA enhances OGT-mediated O-GlcNAcylation of NLRP3, leads to abnormal lipid accumulation, and induces pyroptosis in HepG2 cells	Zhang et al. (2024b)

## O-GlcNAcylation and autophagy

Autophagy is the process by which cells degrade and recycle proteins and organelles to maintain intracellular homeostasis (Liu et al., 2023). While autophagy is crucial for cellular quality control and survival, dysregulation of autophagy, including a lack of coordination between autophagosome formation and lysosomal degradation, can lead to autophagy-dependent cell death (Nah et al., 2022). Previous studies have highlighted the significance of O-GlcNAcylation in cancer initiation and progression through its regulation of autophagy.

Cytotoxic small molecule (SM15) is a small molecule that acts as a potent autophagy inhibitor. SM15 is demonstrated to enhance the O-GlcNAcylation of SNAP29 and inhibits the formation of the SNARE fusion complex, which leads to the blockage of autophagic flux and ultimately results in cell death (Pellegrini et al., 2023). The unc-51-like-kinase 1 (ULK1) complex is an essential regulator of mammalian autophagy, and its function is largely conserved across all eukaryotes, highlighting its significance (Zachari and Ganley, 2017). The O-GlcNAcylation of ULK1 is essential for its binding to and phosphorylation of ATG14L. This process activates the lipid kinase VPS34, which subsequently leads to the production of phosphatidylinositol-(3)-phosphate (PI(3)P). PI(3)P is necessary for phagophore formation and the initiation of autophagy (Pyo et al., 2018). A similar result indicated that ULK1 O-GlcNAcylation at Ser^409^ and Ser^410^ promotes ULK1 stability and autophagosome-lysosome fusion, which could promote HNSCC survival by enhancing autophagy (Shi et al., 2022). In contrast to the findings of the two studies mentioned above, the O-GlcNAcylation of AMP activated protein kinase (AMPK) suppresses autophagic flux by targeting the AMPK-ULK1 pathway in bladder cancer cell lines. This suppression, in turn, promotes the development and progression of bladder cancer (Table 1) (Jin et al., 2020).

Given that Autophagy and O-GlcNAcylation play critical roles in tumors, understanding the specific mechanisms of O-GlcNAcylation in regulating autophagy could provide valuable insights for potential cancer therapies.

## O-GlcNAcylation and ferroptosis

Ferroptosis is a non-apoptotic form of regulated cell death characterized by the lethal accumulation of iron-dependent membrane-localized lipid peroxides (Zhou Q. et al., 2024). Since its discovery in 2012, ferroptosis has been viewed as a potential strategy for cancer treatment (Dixon et al., 2012).

Recent studies have revealed a connection between O-GlcNAcylation and ferroptotic cell death. Yu’s group discovered that inhibiting O-GlcNAcylation enhances both ferritinophagy and mitophagy, leading to increased sensitivity to ferroptosis. Specifically, reduced O-GlcNAcylation promotes the interaction between ferritin heavy chain (FTH) and NCOA4, the receptor for ferritinophagy. This interaction facilitates ferritin degradation, leading to the release of labile iron. The released iron accumulates in the mitochondria, which amplifies lipid peroxidation and promotes ferroptosis (Yu et al., 2022). Transferrin receptor (TFRC) is a key protein that facilitates iron import, which promotes ferroptosis by increasing cellular iron uptake (Hong et al., 2021; Tang et al., 2021). The de-O-GlcNAcylation of TFRC at Ser^687^ has been confirmed to reduce polyubiquitination on Lys^665^, thus enhancing the TFRC protein stability and increasing the ferroptosis sensitivity of HCC cells (Zhou X. et al., 2024). However, Zhu et al. found that O-GlcNAcylation increased the sensitivity of HCC cells to ferroptosis by significantly enhancing the transcriptional activity of YAP and the expression of TFRC (Zhu et al., 2021). These findings illustrate the different roles of O-GlcNAcylation in regulating iron metabolism mutations in HCC. TGF-ZEB1 pathway has been reported to exhibit increased cancer cells susceptibility to ferroptosis (Viswanathan et al., 2017). In pancreatic cancers, glucose-activated O-GlcNAc modification of ZEB1 at Ser^555^ enhances its stabilization and nuclear translocation, thus decreasing lipid peroxidation and ferroptosis in mesenchymal pancreatic cancer cells (Table 1) (Wang et al., 2022). Therefore, targeting O-GlcNAcylation to induce ferroptosis could be a potential therapeutic strategy for ferroptosis-based therapy.

## O-GlcNAcylation and necroptosis

Necroptosis is the result of mitochondrial changes and plasma membrane permeabilization, leading to the release of cytoplasmic contents into the extracellular space and triggering an inflammatory response (Beretta and Zaffaroni, 2022).

During necroptotic cell death, the formation of receptor-interacting protein kinases 1/3 (RIPK1/3) induces the phosphorylation of pseudo kinase mixed lineage kinase domain-like protein (MLKL), leading to cell membrane destruction (Cho et al., 2009; He et al., 2009; Grootjans et al., 2017). Recently, it was reported that OGT-mediated O-GlcNAcylation to be involved in the necroptosis of inflammatory diseases. O-GlcNAcylation of the RIPK3 at Thr ^467^ prevented RIPK3-RIPK1 hetero-and RIPK3-RIPK3 homo-interaction and inhibited innate immunity and necroptosis signaling (Li et al., 2019). Wu-Mei-Wan (WMW) is a classic traditional Chinese herbal medicine that has been one of the key formulations for treating digestive diseases from ancient times to the present (Wu et al., 2020). Wu et al. identified 11 manufacturer compounds in WMW using high-performance liquid chromatography (HPLC). They found that hesperidin, coptisine and ginsenoside Rb1 promoted RIPK3 O-GlcNAcylation by increasing OGT levels and decreasing OGA activity. This process inhibits necroptosis and ultimately helps alleviate TNBS-induced colitis (Wu F. et al., 2021). Similar results observed by Park et al. indicated the protective role of O-GlcNAcylation in Alzheimer’s disease (AD). O-GlcNAcylation can inhibit necroptosis by modifying RIPK3, which alleviates AD pathology, including Aβ accumulation, neuronal loss, neuroinflammation, and microglial dysfunction (Park et al., 2021). In addition, O-GlcNAcylation of RIPK1 inhibits its phosphorylation at Ser^166^ and prevents the formation of the RIPK1-RIPK3 complex, thereby protecting red blood cells (RBCs) from necroptotic cell death (Table 2) (Seo et al., 2023).

While there is no direct evidence that O-GlcNAcylation regulates cancer cells through the mechanisms described above, these studies suggest new directions for targeted cancer therapy.

## O-GlcNAcylation and pyroptosis

Pyroptosis is a type of cell death that is dependent on caspases. It involves the formation of pores in the cell membrane, leading to cell swelling, rupture of the plasma membrane, and the release of all intracellular contents (Huang et al., 2022). p53 is a crucial tumor suppressor, and the loss of p53 function often precedes cancer development (Zhang et al., 2020). Wang et al. show that the de-O-GlcNAcylation of p53 enhances its stability. This increased stability leads to the transcriptional upregulation of genes related to the Bcl-2 family and death receptors, promoting pyroptosis in tumor cells (Table 1) (Wang et al., 2024).

While numerous studies have shown the crucial role of pyroptosis and O-GlcNAcylation in various cancers, research on the interplay between the O-GlcNAcylation and pyroptosis axis in cancer remains limited, as most studies concentrate on certain chronic diseases (Table 2). He et al. recently revealed that O-GlcNAcylation of NEK7 induced by OGT promotes the progression of osteoarthritis (OA) by enhancing chondrocyte pyroptosis through the suppressive interaction between NEK7 and NLRP3 (He et al., 2024). Additionally, lipopolysaccharide (LPS) induces pyroptosis in human gingival fibroblasts (HGFs) by increasing OGT expression, which promotes the O-GlcNAcylation of NLRP3. This indicates that O-GlcNAcylation of NLRP3 was a driving factor for periodontitis (Yang et al., 2024). Another study showed that bisphenol A (BPA) enhances OGT-mediated O-GlcNAcylation of NLRP3, leads to abnormal lipid accumulation, and induces pyroptosis in HepG2 cells, thus accelerating the progression of non-alcoholic fatty liver disease (NAFLD) *in vitro* (Zhang Y. et al., 2024). Thus, studying pyroptosis in other diseases enhances our understanding of the O-GlcNAcylation-pyroptosis axis in cancer.

## Potential therapeutic applications of O-GlcNAcylation-modified programmed cell death

Given the adverse roles of dysregulated O-GlcNAcylation, particularly hyper-O-GlcNAc in cancers (Lu et al., 2022), targeting O-GlcNAcylation to modulate key proteins involved in PCD presents a promising strategy for clinical anti-cancer therapies.

**FIGURE 3:**
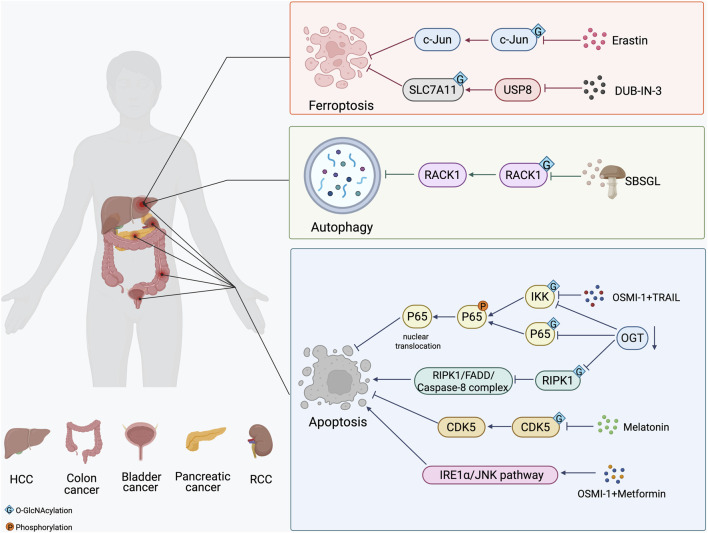
The strategy of O-GlcNAcylation in the treatment of in different cancers. The O-GlcNAcylation of proteins plays a crucial role in cancer development by affecting apoptosis, autophagy and ferroptosis. Small molecule inhibitors of OGT and OGA, several natural and synthetic compounds, or targeting OGT may provide a novel approach to cancer treatment.

Several small-molecule inhibitors have been developed to either directly inhibit the activity of OGT or OGA, thereby manipulating the O-GlcNAcylation of target proteins (Zhang D. et al., 2024). OSMI-1 is a small molecule that acts as a highly specific inhibitor of OGT from the quinolinone-6-sulfonamide (Q6S) class (Very and El Yazidi-Belkoura, 2022). This compound shows potential for therapy, particularly as it can enhance the effectiveness of certain cancer treatments. Inositol-requiring enzyme 1 α (IRE1α), a sensors for ER stress, plays a role in apoptosis through the IRE1α/JNK pathway. Recent studies have shown that combinatorial treatment of colon cancer cell with metformin and OSMI-1 leads to a more pronounced induction of apoptosis. This enhancement occurs through the activation of the IRE1α/JNK pathway, which is facilitated by a reduction in O-GlcNAcylation (Le et al., 2023). In addition, the combination of TRAIL and OSMI-1 shows promise as a therapeutic strategy for overcoming TRAIL resistance in the treatment of colon cancer (Lee S. J. et al., 2021). TRAIL activates NF-κB signaling for survival and growth, which causes resistance to apoptosis. However, when OSMI-1 is introduced, it decreases the O-GlcNAcylation of IkappaB kinase (IKK), inhibiting IKK activity and the downstream signaling pathway. This results in reduced phosphorylation and nuclear translocation of the NF-κB p65 subunit and enhanced apoptosis in cancer cells (Figure 3). Additionally, the cleavage of Gasdermin-D (GSDMD) is crucial for initiating pyroptosis. Treatment with the OGA inhibitor Thiamet-G, which raises global O-GlcNAc levels, alleviates LPS-induced endothelial injury by inhibiting GSDMD cleavage and decreasing markers of pyroptosis, ultimately improving outcomes in sepsis (Yu et al., 2024).

In addition to small molecule inhibitors of OGT and OGA, several natural and synthetic compounds could reduce the proliferation of cancer cells by regulating O-GlcNAcylation modification. Melatonin’s biological functions extend beyond the regulation of the circadian rhythm (Moloudizargari et al., 2021). It is crucial for maintaining glucose homeostasis and energy metabolism. Research has shown that melatonin can inhibit the proliferation of bladder cancer cells and promote their apoptosis by suppressing the O-GlcNAcylation of cyclin-dependent-like kinase 5 (CDK5) and reducing the expression level of CDK5 (Wu J. et al., 2021). The mechanism by which melatonin reduced O-GlcNAc levels of CDK5 involved a decrease in the expression of GFAT, the rate-limiting enzyme of the HBP, leading to a significant decrease in UDP-GlcNAc levels following melatonin treatment. In liver cancer, Ferroptosis inducer erastin inhibits O-GlcNAcylation of c-Jun, decreases its protein expression, transcriptional activity, and nuclear accumulation. c-Jun activity reduction promoted ferroptosis and reduced the malignancy of liver cancer cells (Chen et al., 2019). Ganoderma lucidum, a therapeutic fungus, is a significant target for cancer treatments involving abnormal levels of O-GlcNAcylation. Sporoderm-broken spores of G. lucidum (SBSGL), which contain primarily triterpenoids and polysaccharides, have been shown to effectively inhibit hepatoblastoma malignancy and modulate autophagic flux by decreasing O-GlcNAc modifications in the Receptor for activated C kinase 1 (RACK1) protein and its protein levels. This finding suggests that SBSGL could be a promising complement to conventional therapies (Shen et al., 2024). Additionally, DUB-IN-3, the inhibitor of ubiquitin specific peptidase 8 (USP8), shows effective anti-cancer responses. Mechanistic studies reveal that USP8 stabilizes OGT via inhibiting poly-ubiquitination process on OGT protein, thus increasing the Ser^26^ O-GlcNAcylation of solute carrier family 7, member 11 (SLC7A11) (Figure 3). Thus, pharmacological inhibition of USP8 induces ferroptosis by reducing the stability of OGT and ultimately inhibits the progression of HCC (Tang et al., 2023).

Targeting OGT, OGA, and HBP pathways to modulate PCD may provide a novel approach to cancer treatment. Knocking down OGT has been shown to increase sensitivity to sunitinib in renal cell carcinoma (RCC). Specifically, reduced OGT expression inhibits RIPK1 O-GlcNAcylation and promotes the formation of RIPK1/FADD/Caspase-8 complex, thereby enhancing RIPK1-dependent apoptosis induced by sunitinib (Zeng et al., 2024). In pancreatic cancer, OGT knockdown in PDAC cells leads to a decrease in the O-GlcNAcylation of both IKKα and p65. This reduction is accompanied by lower levels of phosphorylated IKK and p65, decreased nuclear localization of p65, and diminished activation of NF-κB signaling (Ma et al., 2013). Inhibition of NF-κB signaling has been shown to result in PDAC cell apoptosis (Liptay et al., 2003). In addition, the inhibition of OGT, in combination with low-dose chemotherapy, can cause p53-proficient colon cancer cells to switch from senescence to apoptosis (Figure 3). This shift has the potential to enhance the efficacy of chemotherapy for colon cancer while reducing side effects (Loison et al., 2024). Although we have explored some of these important mechanistic details, there is limited research on treating cancer by modulating O-GlcNAcylation. Therefore, integrative mechanisms of O-GlcNAcylation need more studies to identify more targets beneficial to drug research and development.

## Conclusion and perspectives

Since the initial discovery of O-GlcNAcylation in 1984 (Torres and Hart, 1984), significant efforts have been made to uncover the functions and roles of this PTM. Recent evidence highlights the essential role of O-GlcNAcylation in the development of various cancers. However, the impact of O-GlcNAcylation on PCD in cancer remains largely unclear.

Developing tools and approaches to deepen our understanding of O-GlcNAcylation as an epigenetic mark is a major challenge in the field (Dupas et al., 2023). For instance, the development of liquid chromatography-mass spectrometry (LC-MS) has enabled accurate and large-scale prediction of O-GlcNAcylation sites in specific proteins (Xu et al., 2021). The advancement of single-cell isolation and analysis can provide more detailed profiling of individual cell-specific responses, ranging from gene expression to proteomics (Lee B. E. et al., 2021). As a result, the field is poised for rapid discoveries that will further elucidate the mechanisms of O-GlcNAcylation in PCD.

There are still many barriers to the clinical use of OGT or OGA inhibitors. Altering global O-GlcNAcylation levels in cells can impact the O-GlcNAcylation of numerous proteins unrelated to the disease, potentially leading to severe side effects or the development of new conditions (Lu et al., 2022). Moreover, these inhibitors often exhibit high toxicity, low efficacy, poor water solubility, and limited cell permeability, making *in vivo* studies challenging (Yang et al., 2022). Therefore, there is an urgent need for methods that specifically target the modulation of O-GlcNAcylation on PCD proteins for cancer therapy (Zhang D. et al., 2024).

A well-designed nanocarrier can enhance *in vivo* studies in animal models by improving the solubility and cell permeability of certain compounds (Yang et al., 2022). Ge et al. demonstrated the nanobody-fused split OGA, designed to serve as an O-GlcNAc eraser, successfully deglycosylated a broad range of target proteins. It has high selectivity and little effect on overall O-GlcNAc levels (Chen et al., 2024). Dual-specificity (DS) aptamers are modular RNA molecules designed to connect two aptamer motifs through a linker domain. In cells, they induce proximity between OGT and a specific protein, resulting in increased O-GlcNAcylation of the substrate (Zhu and Hart, 2023). Additionally, a noteworthy strategy involves using short peptides that contain glycosylation sites to competitively inhibit glycosylation in specific proteins (Zhu et al., 2023). This approach opens up future opportunities for the development of targeted drug therapies. While these technologies are relatively new and the pathway to put them into clinical practice is long, O-GlcNAcylation-regulated PCD will provide novel targets for cancer treatment.

## Glossary

O-GlcNAc: O-linked β-N-acetylglucosamine
PCD: programmed cell death
PTMs: protein post-translational modifications
HBP: hexosamine biosynthetic pathway
UDP-GlcNAc: uridine diphosphate N-acetylglucosamine
UTP: uridine triphosphate
UDP-GlcNAc: UDP-N-acetylglucosamine
GlcNAc: N-acetylglucosamine
OGT: O-GlcNAc transferase
OGA: O-GlcNAcase
ncOGT: nucleocytoplasmic OGT
mOGT: mitochondrial OGT
sOGT: short OGT
TPR: tetratricopeptide repeat
GH84: glycoside hydrolase (GH) family 84
ER: endoplasmic reticulum
STAT3: signal transducer and activator of transcription 3
FOXO1: forkhead box protein O 1
TRAIL: tumor necrosis factor (TNF)-related apoptosis-inducing ligand
ULK1: unc-51-like-kinase 1
PI(3)P: production of phosphatidylinositol-(3)-phosphate
AMPK: AMP activated protein kinase
TFRC: transferrin receptor
HCC: hepatocellular carcinoma
RIPK1/3: receptor-interacting protein kinases 1/3; mixed lineage kinase domain-like protein
WMW: Wu-Mei-Wan
AD: Alzheimer’s disease
RBCs: red blood cells
LPS: lipopolysaccharide
HGFs: human gingival fibroblasts
BPA: bisphenol A
NAFLD: non-alcoholic fatty liver disease
Q6S: quinolinone-6-sulfonamide
GSDMD: Gasdermin-D
CDK5: cyclin-dependent-like kinase 5
SBSGL: sporoderm-broken spores of G. lucidum
RACK1: receptor for activated C kinase 1
USP8: ubiquitin specific peptidase 8;
SLC7A11: solute carrier family 7, member 11
RCC: renal cell carcinoma
LC-MS: liquid chromatography-mass spectrometry
POI: protein of interest
