# Correction: Insights into O-GlcNAcylation and programmed cell death in cancer

**DOI:** 10.3389/fcell.2025.1712372

**Published:** 2025-10-23

**Authors:** Frontiers Editorial Office

**Affiliations:** Frontiers Media SA, Lausanne, Switzerland

**Keywords:** O-GlcNAcylation, cancer, programmed cell death, apoptosis, autophagy, pyroptosis, ferroptosis, necroptosis

Author **Wenhao Ren** was erroneously omitted as equal contributing author, and was erroneously assigned as a corresponding author.

Author **Jingjing Zheng** was erroneously omitted as the first corresponding author, and was erroneously assigned as an equally contributing author.

The original version of this article has been updated.

